# The Role of Resilience on Correctional Worker Wellbeing: A Systematic Review

**DOI:** 10.1192/j.eurpsy.2025.281

**Published:** 2025-08-26

**Authors:** S. Lalji-Mawji, P. Harris, W. M. Tomlin, M. O. Ahedor, B. Ostemeyer, A. T. Olagunju

**Affiliations:** 1 Michael G. DeGroote School of Medicine; 2 Psychiatry and Behavioural Neurosciences, McMaster University; 3 Forensic Psychiatry Program, St. Joseph’s Healthcare Hamilton, Hamilton, Canada; 4 Psychiatry and Behavioural Neurosciences, University of Oklahoma, Oklahoma, United States; 5 Psychiatry, University of Adelaide, Adelaide, Australia

## Abstract

**Introduction:**

Correctional workers face uniquely stressful conditions that can impact their personal and professional wellbeing. Resilience, defined as the ability to adapt and thrive in adverse conditions, may be a key mitigator of occupational stress. Despite its potential benefits, few reviews examine the wellbeing of correctional workers (Miller, O., Bruenig, D., & Shakespeare-Finch, J. 2022; 49(11), 1559-1579) but have not comprehensively addressed resilience.

**Objectives:**

1. Describe resilience and summarize measures used to assess resilience in correctional settings. 2. Investigate the role of resilience on psychosocial wellbeing, burn-out, work performance, work leaves, attitudes, response to adverse incidents and turn-over among correctional workers. 3. Describe risk factors associated with resilience among correctional workers. 4. Describe study-defined strategies to build resiliency and relevant recommendations for future research and clinical practice.

**Methods:**

The present review was conducted in accordance with Preferred Reporting Items for Systematic Reviews and Meta-Analyses (PRISMA) guideline. Major databases (PubMed/MEDLINE, Embase, PsycINFO, Scopus, and CINAHL) were searched for eligible reports. At least two independent reviewers were responsible for screening and data collection. Conflicts were resolved via discussion, with input from senior authors when necessary. Quality appraisal was conducted for all included reports.

**Results:**

As shown in Figure 1, a total of 679 articles were identified through major database searches. Title and abstract screening yielded 51 articles eligible for full-text review. The majority of articles were set in North American correctional facilities. Key factors for resilience included support systems, purpose, and optimism, while workplace adversity was a risk factor. Resilience was found to reduce symptoms of burnout and depression, and be crucial for managing psychosocial wellbeing. The presentation will discuss strategies to build resilience and highlight relevant recommendations.

**Image 1:**

**Figure:**
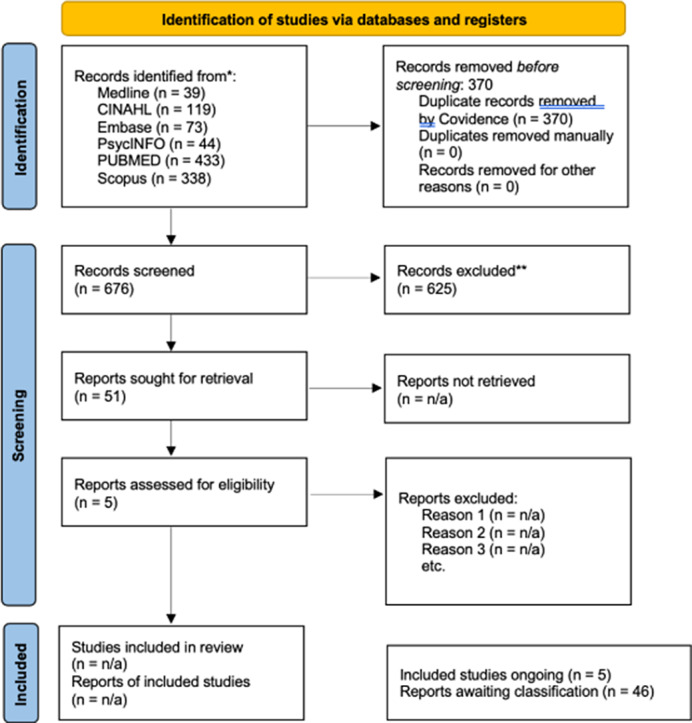

**Conclusions:**

Overall, resilience was found to play a mediating role in the wellbeing of correctional workers. Further studies involving a standardized measure of resilience and broader populations and correctional settings are required to improve the validity and generalizability of findings.

**Disclosure of Interest:**

None Declared

